# ASO Author Reflections: Area Socioeconomic Status is Associated with Refusal of Recommended Surgery in Patients with Metastatic Bone and Joint Disease

**DOI:** 10.1245/s10434-024-15517-0

**Published:** 2024-06-09

**Authors:** Mitchell S. Fourman, Kyle Mani

**Affiliations:** 1 Department of Orthopaedic Surgery, Montefiore Einstein, Bronx, NY USA; 2 Albert Einstein College of Medicine, Bronx, NY USA

## Past

Cancer care quality and outcomes have been well-associated with patient socioeconomic status.^1^ Factors such as family responsibilities, job security, food availability, and transportation can significantly impact the ability for a patient to comply with cancer treatment and surveillance recommendations. Given the multifactorial and community-dependent nature of socioeconomic deprivation, multivariate rating scales such as the Yost Index or Area Deprivation Index (ADI) have been used to ‘rank’ community vulnerability. These scales provide a pooled objective value, attempting to avoid the inevitable confounding effects that including only a select number of sociodemographic factors in an analysis can result in. These pooled indices are a powerful representation of the patient’s home and community support over the course of their care, and higher degrees of community socioeconomic deprivation have been independently associated with worse treatment outcomes^2^ and limited postoperative functional recovery.^3^

## Present

The present work leveraged the Surveillance, Epidemiology, and End Results (SEER) database of the National Cancer Institute to evaluate the influence of socioeconomic vulnerability, measured using the Yost Index, on the outcomes of patients with *de novo* metastatic cancer to bone in whom surgery was recommended.^4^ Of 14,943 surgical candidates treated from 2010 to 2018, 19.6% refused surgery. Independent predictors of refusal were advanced age, African American race, single patients, and those in the lowest Yost Index quintile. Interestingly, the anatomy of the surgical lesion and cancer-specific factors did not independently influence surgical decision making, although it should be noted that, in general, SEER does not provide a substantial amount of anatomic and surgical granularity within its dataset.

The findings of the present work emphasize the importance of more strongly emphasizing personal and community factors over medical factors when discussing potential interventions for patients with cancer. They also suggest that patients with greater degrees of social support (i.e., single vs. married status) are more likely to accept surgical interventions for metastatic cancer to bone.

## Future

While the body of literature on the influence of socioeconomic factors on cancer care outcomes has increased substantially over the past few years, we are still at an early stage in our understanding of which individual and community-level factors most strongly influence these outcomes. Of particular importance is to best understand the influence of race on cancer surgical decision making beyond a surrogate for socioeconomic disadvantage. Distrust of medicine and medical institutions must be considered. Interventions to improve care outreach and transparency should be the cornerstone of efforts towards making inroads with historically disadvantaged populations. Webb Hooper et al.^5^ suggest that participant-driven dialogs on oncology care can lead to substantially improved interactions with and trust in cancer center care. The present work should serve as additional evidence that community-level, culturally sensitive interventions are necessary to broadly improve cancer care outcomes.
